# Recommendations for the Establishment and Operation of Human Milk Banks in Europe: A Consensus Statement From the European Milk Bank Association (EMBA)

**DOI:** 10.3389/fped.2019.00053

**Published:** 2019-03-04

**Authors:** Gillian Weaver, Enrico Bertino, Corinna Gebauer, Anne Grovslien, Radmila Mileusnic-Milenovic, Sertac Arslanoglu, Debbie Barnett, Clair-Yves Boquien, Rachel Buffin, Antoni Gaya, Guido E. Moro, Aleksandra Wesolowska, Jean-Charles Picaud

**Affiliations:** ^1^Human Milk Foundation Harpenden, United Kingdom; ^2^Neonatal Unit of Turin University City of Health and Science of Turin, Turin, Italy; ^3^Abteilung Neonatologie Klinik und Poliklinik für Kinder und Jugendliche Leipzig, Germany; ^4^Neonatal Unit Milk Bank, Oslo University Hospital, Oslo, Norway; ^5^First Serbian Milk Bank Institute of Neonatology, Belgrade, Serbia; ^6^Division of Neonatology Department of Pediatrics, Istanbul Medeniyet University, Istanbul, Turkey; ^7^Greater Glasgow and Clyde Donor Milk Bank Royal Hospital for Sick Children, Glasgow, United Kingdom; ^8^PhAN Institut National de la Recherche Agronomique (INRA), Université de Nantes, CRNH-Ouest, Nantes, France; ^9^Neonatal Intensive Care Unit Hopital de la Croix-Rousse, Hospices Civils de Lyon, Lyon, France; ^10^Banc de Teixit F. Banc de Sang i Teixits de les Illes Balears, Institut d'Investigacio Sanitaria Illes Balears (IdISBa), Barcelona, Spain; ^11^Italian Association of Human Milk Banks (AIBLUD) Milan, Italy; ^12^Department of Neonatology Faculty of Health Science, Medical University of Warsaw, Warsaw, Poland; ^13^Laboratory of Human Milk and Lactation Research Holy Family Hospital Regional Human Milk Bank, Warsaw, Poland; ^14^CarMeN Unit INSERM U1060, INRA U1397, Claude Bernard University Lyon 1, Pierre Bénite, France

**Keywords:** donor human milk (DHM), Human Milk Bank (HMB), breastfeeding, pasteurization, donor screening, bacteriology testing

## Abstract

**Objectives:** To develop recommendations from the European Milk Bank Association (EMBA) for the establishment and operation of human milk banks (HMB) in Europe.

**Method:** A working group comprising members of the EMBA was convened in 2015 to develop Europe-wide recommendations for milk banks. Each member had experience of guideline development and/or milk banking operations. An initial survey was agreed using collated published global recommendations. A total of 108 potential recommendations were included in the survey; responders noted which were included in their national guidelines. The responses were collated, compared, and discussed and the group determined where there was consensus and where substantial or minor differences were identified. Where there was consensus or robust published evidence on which to base recommendations these were included. When there was no consensus and no clear evidence base, a statement of explanation based on collective expert opinion was agreed.

**Results:** Published, internationally available guidelines with recommendations for human milk banks from France, Italy, and the UK, together with guidelines from Austria, Denmark, Germany, Norway, Slovakia, Spain, Sweden, and Switzerland were included as source materials. These covered: General recommendations; Donor recruitment and screening; Expression, handling, and storage of donor human milk (DHM); Pooling of DHM; Milk screening; Milk treatment (pasteurization); Delivery of DHM to recipients.

**Conclusions:** Evidence based recommendations and consensus statements from the EMBA will now be published on the EMBA website to assist in the safe establishment and operation of HMBs throughout Europe. These have also been used to inform the chapter on human milk to be included in the 2019 edition of the *Guide to the quality and safety of tissues and cells for human application*, published by the European Directorate for the Quality of Medicines & HealthCare (EDQM).

## Background

Human milk (HM) is the preferred nutrition for preterm infants (1), but not all mothers are able to provide their child with enough milk. There are specific beneficial effects of breastfeeding in these infants as HM feeding reduces the risk of short term and long term complications related to prematurity. Donor HM (DHM) can supplement the supply of maternal breastmilk when it is insufficient or provide the preferred alternative when the mother is not breastfeeding. DHM is beneficial for the health of preterm infants, first because significant properties are preserved following holder pasteurization (2) and secondly because it prevents feeding these infants with a preterm formula. According to the World Health Organization (1) the American Academy of Pediatrics (3) and the European Society of Pediatric Gastroenterology, Hepatology, and Nutrition (4) the feeding of preterm infants with mother's own milk is recommended as the first choice and if this is not available pasteurized DHM from an established milk bank should be the next alternative.

The main benefits for preterm infants that receive DHM instead of formula are faster gastric emptying, faster attainment of full enteral feedings, improved gut growth and maturation, decreased risk of necrotizing enterocolitis and late onset sepsis, improved neurodevelopmental outcomes, less retinopathy of prematurity and improved visual development (5–9).

HMBs have existed since 1909 in Europe and countries that supported the development of a significant number of HMBs developed national guidelines to harmonize practices. The European Milk Bank Association^1^ (EMBA) was officially launched in October 2010. According to the definitions agreed by the EMBA Board of Directors:

- Human milk banks (HMB) collect, screen, store, process, and distribute DHM.
- DHM is breastmilk that has been expressed by a mother and provided freely to a HMB to be fed to another mother's child.

The EMBA constitution is available to view on the EMBA website ^1^. The Association welcomes membership from HMBs, milk bank associations and individuals who support the aims of the Association. Membership affords the opportunity to access conference presentations from the past 6 years as well as additional materials of interest to those who bank or use DHM.

The EMBA is a non-profit organization that was established to promote milk banking and to encourage international co-operation between the HMBs of the countries of Europe as well as globally. The association's headquarters are in Milan, Italy but association meetings and conferences are held in different locations within Europe. The primary aim of the EMBA is to support and promote exclusive breastfeeding, which is universally acknowledged to be the optimal method of feeding all infants. The use of DHM from HMBs for preterm and sick infants temporarily without access to their own mother's milk has been widely shown to be linked to increased rates of exclusive breastfeeding on discharge from neonatal units (10, 11).

The EMBA was also established to support human milk banking in Europe by promoting safe practices and fostering sustainable operations. Hence a further aim of the association, as listed in the EMBA Constitution ^1^ is to “Prepare international and regularly revised guidelines to set standards for the practice of milk banking.” The usual methodology for establishing guidelines is to convene appropriate multidisciplinary expertise, consider all of the stages of a process and conduct a review of the evidence before making recommendations linked to the strength of the evidence. In the absence of such a published guideline, aimed at milk banks throughout Europe, it is expected that the development of Europe-wide recommendations by EMBA for HMBs will promote best practice, optimize quality, and safety within the HMBs that are currently operational as well as offer valuable assistance to those who are establishing a new HMB. Evidence and expert opinion based recommendations should be the standard throughout Europe to promote consistent practices both within and between countries.

There are currently 226 HMBs operating in 28 countries in Europe. These include the first milk banks in Estonia, Lithuania, Poland, Portugal, and Russia. A further 16 HMBs are planned. The current status of HMBs in Europe is maintained on the EMBA website ^1^.

The 226 HMBs in Europe incorporate newly established organizations with a limited history of operational expertise as well as ones that have links to the very early milk banks founded over a century ago. They include national and regionalized services as well as small operations serving a single neonatal unit. The volumes of DHM that are tested and processed and provided mainly to sick and preterm infants vary widely from country to country ^1^ as well as within a single country (12) as do the criteria for receipt of DHM. An EMBA coordinated survey of all the HMBs in Europe will be carried out in 2019. This will gather up to date information about the extent of milk banking activity including the number of donors recruited, volumes of milk donated and processed, volumes of milk provided to hospitals, and the number of recipients. It will also collate further information about milk banking practices.

There are currently no published Europe-wide guidelines however internationally published guidelines for HMBs are freely available from France^2^, Italy (13), and the UK (14). Countries with nationally agreed and recognized guidelines include Austria, Denmark, Germany, Norway, Slovakia, Spain, Sweden, and Switzerland. English translations of recently updated (2017–2018) national guidelines from Sweden and Spain can be downloaded via links from the EMBA website. Differences exist between national guidelines as a result of variations in practices, regulation, and organization of HMBs in each country. Differences in practices are due to the lack of evidence for some points related to the operation of a HMB. DHM status globally is variable; it is either considered a food, a health product food or a tissue; the latter ones being subject to stricter regulation. Organization of HMBs is different when collecting and delivering DHM only for infants hospitalized in the same building, or over a more or less wide area including several health care institutions.

The HMBs of Europe also have very different historical backgrounds ^1^ and in most cases have operated independently of each other although regular meetings take place between milk banking practitioners in Norway and Sweden and between those of Austria, Germany, and Switzerland.

A major role of the EMBA has, since its inception, been the provision of education, largely via the organization of international congresses. Presentations at these congresses have revealed similarities between the milk banking practices throughout Europe and have also revealed fundamental differences. These have substantiated the results of surveys of HMBs carried out both before and after the establishment of EMBA in 2007 and in 2012. In some cases the official national or local recommendations from various countries lead to significant differences in practice. For example most published guidelines recommend that all DHM should be heat treated [using the standard holder pasteurization method^2^ (13, 14)]. However, in Norway mainly raw (non-heat treated) DHM is provided by the country's 12 milk banks. This is considered possible and reasonable because (1) the demand can be met due to very high breastfeeding rates, even with extremely strict donor screening, (2) there is complete traceability of milk from donor to consumer, (3) there is a small population with very low HIV and hepatitis rates, (4) extremely rigorous and regularly repeated testing of donors (every 3 months) is practiced (15, 16). However, in almost half of the 20 official HMBs in Germany both raw and pasteurized DHM is available and used in accordance with local clinical judgment based on the gestation and health of the recipient (17).

## Objective

One of the clear objectives of the EMBA ^1^ has been to develop Europe-wide recommendations for HMBs. This is especially needed by countries without experience of human milk banking or the use of DHM. Such recommendations should optimize safety and draw upon the available evidence. The processes and practices within human milk banking do not lend themselves to randomized controlled trials and there are few meta-analyses or systematic reviews available to refer to. In the absence of these, expert opinion is required and where this differs, a consensus and in some cases compromise is sought. The EMBA Guideline Working Group was formed in 2015 to undertake this task. EMBA members from 13 countries contributed to the work of the group. Throughout its 3 year existence, members of the Guideline Working Group have included representatives from Austria, France, Germany, Italy, Norway, Poland, Portugal, Serbia, Slovenia, Slovakia, Spain, Switzerland, and the UK.

## Method

The three main tasks of the group were:

1. to complete a comprehensive survey of current practices within their national milk banks (See Appendix for details of the 108 survey questions)
2. to use the known guidelines to assess where consensus could be achieved
3. identify where research evidence is available to support recommendations.

A useful tool in developing the structure for the survey was the global implementation framework (18) published by the NGO PATH^3^. The framework includes a compilation of practices in HMBs from donor recruitment to delivery of DHM to the recipient.

Each of the group members completed the survey in accordance with their local/regional or national guidelines and, if not included in the guidelines, in accordance with local practice. Once completed, the survey responses were collated and highlighted according to whether international consensus was apparent, whether there was near consensus and where practices and recommendations differed significantly. The next step was to note where no published evidence was available. A list of agreed recommendations that were evidence based and that could form the basis of EMBA recommendations was drawn up and presented to the wider EMBA membership at further meetings. Where there was no consensus and no evidence upon which to determine a recommendation, expert opinion within the group was used to decide whether to include a recommendation or to provide an explanation of why no clear recommendation could be made at that time.

## Results

The recommendations that were found to be included in all of the published guidelines or accepted as best practice according to the available evidence or expert opinion and have therefore been agreed by the Working Group are presented below. Many of these are underpinned by principles of good manufacturing process (GMP) especially those related to consistency and quality control.

### EMBA Recommendations for the Establishment and Operation of Human Milk Banks in Europe

#### General Recommendations

1. A robust quality assurance plan (e.g., HACCP—Hazard Analysis Quality Control Points) should be in place to ensure the safe operation of the HMB (13, 14).
2. All equipment should be maintained in accordance with manufacturers' instructions and checked and qualified annually to ensure conformity with recommendations (GMP).
3. Containers should be closed or sealed in accordance with manufacturers' recommendations (GMP).
4. Containers should not be overfilled as DHM will expand when frozen (GMP).
5. Containers of human milk should at all times be labeled with the donor's name or unique ID and the date the milk was expressed. In addition, depending on the stage in the process, DHM should also be labeled with the name of the HMB, whether the milk is raw or processed, the milk's expiry date and if the milk is ready (cleared) to use (14).
6. HMBs should at all times minimize exposure of the human milk to sunlight and/or phototherapy lights.
7. HMB staff should have health checks when employed and be immunized in accordance with local hospital or national health service protocols (14).
8. Staff should undertake training by an experienced member of staff (or in accordance with national accredited training programs where available) before undertaking unsupervised work in a HMB (14).
9. DHM should be handled hygienically and HMB staff should wash their hands in accordance with local protocols prior to entering clean areas, handling DHM or equipment used in the collection, storage, testing and processing of DHM (GMP).
10. HMB staff should at all times consider the ethical implications of their work with donors, parents, carers, and infants. For example donors should not be encouraged or coerced to donate more milk than may be optimal for themselves or their infants. The use of DHM should not undermine or interfere with the mother's provision of her own milk or of breastfeeding unless there are concerns about its safety.
11. Records should be kept of all donors and their donations of milk including volumes, dates and any additional relevant information (traceability).
12. Prioritization of recipients should be locally determined (14).
13. Steps should be taken to prevent the temperature of DHM rising during transport e.g., by the use of insulated transport containers (boxes or bags) and thermal (ice) packs or the use of dry ice where necessary (14).
14. The use of tamper evident transport containers is recommended and the temperature of the interior of the container should be monitored during transport using data loggers or checked on arrival. A record of this should be maintained as part of the HMB records (GMP).
15. Transport boxes/bags in which containers of DHM are carried should be insulated and easy to clean.
16. The transport container should be decontaminated between batches of milk. Keep raw milk from different mothers separate. Avoid using the same transport container to transport raw and pasteurized DHM.
17. Containers of DHM should not be placed directly into the transport container—use of clear polythene bags helps the HMB identify the contents and check that they are correctly labeled without the need for unnecessary handling.

#### Donor Recruitment and Screening

1. Recruit donors using clear, non-technical language in printed and digital media.
2. Screening of donors should include both an oral interview and completion of a health questionnaire.
3. Inform prospective donors that they will be required to undertake serological testing.
4. Exclude prospective donors if they:
  - Smoke cigarettes or use nicotine containing products including “vaping,” gums, and other products
  - Use any recreational or “street” drugs.
  - Are known or found to be infected with HIV, hepatitis B or hepatitis C (13, 14)^2^. Additional screening for HTLV (human T-lymphotropic virus), syphilis, and other viral and bacterial infections may be screened for according to local evaluation.
  - Use medications other than those on the EMBA approved medication list ^1^.
  - Have received a recent blood transfusion, tattoo or piercing, or needle stick injury. The. determination of “recent” should be locally determined in accordance with blood donation/transfusion services and methods used for serology testing.
  - Follow a vegan diet without supplementation with Vitamin B12.
  - Have a sexual partner who has, or is at risk of acquiring, sexually transmitted infections.
5. There is no consensus on safe amounts of alcohol consumption prior to expressing human milk for donation. EMBA recommends therefore that donors avoid alcohol and never donate milk expressed whilst they are under the influence of alcohol or are likely to secrete human milk containing alcohol (within 4 h of moderate drinking). Local guidelines on alcohol consumption by breastfeeding mothers should also be considered.
6. Donors should inform the HMB if there are any changes in their behavior or health status that affects their answers to questions related to any of the above.
7. Before accepting a donor's milk, receive written informed consent for its use in accordance with the HMB's protocols including for approved research if relevant.
8. Train all new donors in hand washing and hygiene requirements for expressing, handling, storing, cooling, freezing, and transporting human milk (GMP).
9. Provide appropriate ongoing support for all donors, including where possible, those rejected by the HMB.
10. Provide additional training and support for donors who repeatedly donate milk that does not meet the microbiological or other testing criteria.
11. Do not exclude bereaved mothers from donating their breastmilk if they meet the donor recruitment and screening requirements (19).
12. Once recruited, exclude donation of HM on a temporary basis in the case of any of the following:

– the presence of mastitis
– temporary use of some pharmacologically active substances^1^^,^^4^
– the presence of acute infectious diseases and skin diseases such as herpes simplex or varicella zoster
– fungal infection of the nipple, areola, mammary or thoracic region.

The extent of the temporary deferral of donors will vary according to the duration of the circumstances and medical advice should be sought to exclude the possibility of acceptance of contaminated, unsuitable, or suboptimal milk.

#### Expression, Handling, and Storage of Human Milk for Donation to the HMB

1. Advise donors to collect expressed milk rather than drip milk (14).
2. Accept hand expressed, manual pump-expressed, and electric pump expressed milk.
3. Advise donors to ensure all breast pump equipment is cleaned and disinfected in accordance with manufacturer's recommendations or local hospital protocols if different (GMP).
4. Emphasize the importance of good hygiene and hand washing at all times when communicating with donors about expressing their milk.
5. Discourage the sharing of breast pumps outside of hospital and the use of second hand or pumps on loan unless by a hospital or health care provider.
6. Request that donors freeze milk for donation as soon as possible but within a maximum of 24 h (48 h if collected and stored in hospital refrigerator/freezer).
7. Only containers provided by or approved by the HMB should be used by donors
8. Ensure donated milk is checked for labeling compliance prior to collection or on arrival at the HMB if delivered by the donor.
9. On its arrival at the milk bank, place donated human milk in a suitable holding freezer (freezer for raw milk only and maintaining −20°C).
10. All refrigeration, freezing, and other chilling equipment should only be used for human milk.
11. Monitor and record refrigeration and freezing equipment continuously or at least every 24 h.
12. Store raw and pasteurized human milk in separate clearly labeled refrigerators and freezers or if not possible in separate clearly labeled fridge and freezer compartments.
13. Thaw frozen raw human milk in a refrigerator to prevent its temperature rising above 8°C or if impractical because of time constraints on a counter where it can be monitored and transferred to a refrigerator immediately once thawed.
14. Pooling of DHM.
  14.1 The pooling of DHM from the same donor is acceptable prior to any heat processing
  14.2 Maintain records of all pooling including the names/ID numbers of the donors, dates the milk was expressed and any medications taken.

#### Milk Screening; Pre- and Post-pasteurization Testing

Within Europe, as well as globally, there is no consensus for recommendations for the microbiological testing of DHM either before or after pasteurization (see Table 1). Throughout Europe, local and national guidelines vary both in the timing and frequency of testing and in the acceptance criteria. There is a lack of published evidence to inform decision making and the working group members concluded that to maximize safety of vulnerable immune-compromised recipients, best practice suggests:

- Before pasteurization
  – All pools of milk be tested prior to pasteurization
  – Each batch of milk be tested after pasteurization
  – Acceptance criteria: 10^5^ cfu/ml or less of non-pathogenic organisms and no pathogens for each pool of milk tested prior to pasteurization. Discard all samples of milk from a pool that does not meet this standard.
- After pasteurization
  – Discard the batch if there is any microbial growth detected in a random sample taken after pasteurization.

**Table 1:** Published microbiological screening criteria for acceptance of donor human milk prior to and after pasteurization.

**Guideline**	**Pre-pasteurization: Total confluent bacterial growth**	***Pre-pasteurization: Additional criteria***	**Post-pasteurization**
France: Bonnes pratiques des lactariums^2^	Total (aerobic) flora ≤ 10^6^ CFU/mL	*Staphylococcus aureus* ≤ 10^4^ CFU/mL	Any microbial growth
Italy: Guidelines for the establishment and operation of a donor human milk bank (13)	≤ 10^5^ CFU/mL	*Enterobacteriaceae or Staphylococcus aureus* ≤ 10^4^ CFU/mL	≤ 10 CFU any organism
Sweden: Guidelines for the use of human milk and milk handling in Sweden ^1^	Total aerobic bacteria: No upper limit	• Any pathogenic bacteria from the following‘: • Betahemolytic Streptococci group A, C, or G, Streptococci group B, Listeria, or Salmonella <10^4^ CFU/ml • *Enterobacteriaceae* • *Staphylococcus aureus* • *Pseudomonas aeruginosa* or other Pseudomonas species • *Stenotrophomonas maltophilia* • Acinetobacter species	Total aerobic count ≤ 10 CFU/ml. No pathogenic bacteria are accepted
United Kingdom: NICE Clinical Guideline 93. Donor milk banks; service operation (14)	≤ 10^5^ CFU/mL	*Enterobacteriaceae or Staphylococcus aureus* ≤ 10^4^ CFU/mL	≤ 10 CFU any organism
Australia: Best practice guidelines for the operation of a donor human milk bank in an Australian NICU (20)	≤ 10^5^ CFU/mL	Any *Enterobacteriaceae, Enterococci* or potential pathogens capable of producing heat-stable enterotoxins	Any microbial growth
United States of America (HMBANA): Guidelines for the Establishment and Operation of a Donor Human Milk Bank 2018^a^	Pre-pasteurization testing not included in the recommendations	Pre-pasteurization testing not included in the recommendations	“Any bacteriological growth is unacceptable for heat-processed milk”

a *Human Milk Bank Association of North America. http://hmbana.org*.

It is recognized that most published guidelines include less stringent screening criteria (Table 1) as part of the overall recommendations. The EMBA therefore recommend that where milk banks follow nationally or locally agreed guidance, alternative microbiological screening criteria may be adopted if done so as part of the overall adherence to the guideline being followed.

#### Milk Treatment (Pasteurization)

1. Current recommended heat treatment/pasteurization temperature and time is 62.5°C for 30 min followed by rapid cooling to at least 10°C and preferably 4°C prior to transfer to a freezer (2).
2. Monitor the process and record temperatures throughout the treatment (2).
3. Pasteurized milk that has been freeze dried and vacuum packed if performed in a milk bank in accordance with GMP and all relevant safety procedures may be used.

#### Delivery of DHM to Recipients

1. Never use a microwave oven to defrost or warm milk.
2. All donor milk and containers should be labeled at each stage to ensure traceability and tracking of the milk.
3. The receiving hospital should record/document how donor milk is used including in the infant's hospital notes.
4. Before administration of donor milk, informed consent is required from recipient's mother/parent/carer in accordance with local protocols.
5. It is recognized that some religious beliefs and practices influence the acceptability of anonymized donor human milk and these should be taken into account when discussing donor milk with donors and with parents and carers and when drawing up local protocols.

## Discussion

The Working Group was able to arrive at a consensus for recommendations covering most of the major processes involved in recruiting and screening donors, storing, handling, and transporting DHM and in its testing and processing. In these cases, the evidence, as referenced in the relevant national guidelines was checked and noted. Where published evidence was not available, expert opinion was the basis for the recommendation. The group also highlighted several areas for which consensus could not be achieved and where there was no clear evidence to inform a recommendation. In these instances the group provided a statement of explanation and suggested best practice. The group also acknowledged that not all countries that have milk banks were represented within the guideline group. A further consideration was that HMBs are funded in different ways and have varying resources. It was agreed that to make a single recommendation that may undermine a milk bank's ability to continue to operate would not be in the best interests of the overall population of recipients.

The process by which DHM is tested to provide microbiological safety is an example of where no consensus could be found. Very divergent practices are currently in place throughout Europe and there is no agreement within the published guidelines as to the optimal testing regime either before or after pasteurization or both. There is also no clear evidence base on which to determine the recommendations. However, it is possible to make a recommendation as to which practice will offer optimal safety for all recipients, including the most vulnerable neonates, and through which no untested milk reaches the recipients and the strictest published acceptance criteria are used. Recommended testing regimes exist in Europe that include less frequent sampling [e.g., only testing the first donation and randomly testing subsequent donations as in the Italian guidelines (13)]. There are also recommendations in which the acceptance criteria are less strict (e.g., pre-pasteurization criteria of 10^5^cfu/ml or less of any organisms and 10^4^cfu/ml or less of *Staphylococcus aureus* and *Enterobacteriaceae* and the presence of <10 cfu/ml of any organism post-pasteurization as per the UK's NICE guideline (14). These are acceptable alternatives but do not provide the same assurance of safety. It should be noted however that less strict testing regimes will lead to more processed milk being available. An additional consideration is that if DHM is fed without any heat treatment (as occurs in some parts of Europe), stricter local microbiological testing protocols should be adopted to increase the safety of the recipients.

The tracking of DHM and the traceability of the milk throughout all the HMB processes is an essential component of safety and quality assurance. This is enhanced by the availability of customized and purpose developed barcode tracking systems and the use of the internationally agreed coding system, ISBT128 (21). Regular strict quality control of pasteurization equipment is necessary to maintain optimum safety and quality of DHM (2, 22, 23).

Whilst safety is at the forefront of all recommendations pertaining to HMBs and the use of DHM it is important to ensure that the ethical considerations of banking and sharing human milk and duties of care to the donors as well as the recipients are not overlooked (24).

A limitation of this consensus statement is that no recommendations were made regarding the analysis of HM. HM analysis is a means of assessing protein and energy contents and to facilitate targeted fortification of HM (i.e., to fortify milk according to its individual composition to reach a target composition as a means of helping to cover the theoretical nutritional needs of each infant). Some HMBs include the assessment of HM composition in their practice. This is done either to perform targeted fortification or to select HM with the highest nutrient content for the smallest babies. However, there is no consensus about the best strategy for fortification. Only adjustable fortification, which does not require HM composition assessment, has been shown to improve postnatal growth.

## Conclusions

There are no Europe-wide guidelines covering practices within HMBs. Historically national and/or local guidelines have been developed and although the recommendations include many similarities, clear differences also exist. Developing recommendations for HMBs operating throughout Europe was a challenging task because of the diversity within European countries. However, with the input of leading milk banking experts from a wide range of countries and with additional help from the EMBA Board it was possible to agree a pragmatic approach to where differences could not be resolved through reference to published research. The resultant guidance will now be freely available on the EMBA website to assist in the safe establishment and operation of HMBs throughout Europe. The recommendations have also been used to help inform the chapter on human milk to be included in the 2019 edition of the *Guide to the quality and safety of tissues and cells for human application*, published by the European Directorate for the Quality of Medicines & Health Care (EDQM).

## Author Contributions

GW, EB, CG, AnnG, RM-M, SA, DB, C-YB, RB, AntG, GM, AW, and J-CP contributed to the working group and/or to the discussions of the findings of the working group and all offered amendments to the previous drafts and/or agreed the manuscript.

### Conflict of Interest Statement

The authors declare that the research was conducted in the absence of any commercial or financial relationships that could be construed as a potential conflict of interest. The reviewer MG declared a past co-authorship with one of the authors AG to the handling Editor.

## Footnotes

^1^http://europeanmilkbanking.com

^2^http://ansm.sante.fr/Activites/Elaboration-de-bonnes-pratiques/Bonnes-pratiques-des-lactariums

^3^PATH: http://www.path.org

^4^http://ukamb.org/medication-and-breastfeeding
