# Supplementary material for: Recommendations for the Establishment and Operation of Human Milk Banks in Europe: A Consensus Statement From the European Milk Bank Association (EMBA)
Source: Front Pediatr. 2019 Mar 4;7:53. doi: 10.3389/fped.2019.00053 (PMC6409313; doi:10.3389/fped.2019.00053)
Supplement: Supplementary file 1 [file Data_Sheet_1.docx]

Appendix: EMBA milk bank recommendations survey

The table below includes the questions asked in the survey together with the collated responses from the participants. The questions were derived from the compilation of practices in HMBs from donor recruitment to delivery of DHM to the recipient as collated from global milk banking guidelines by PATH and published in Strengthening Human Milk Banking; a global implementation framework^20^

C = consensus across all responses

SC = some consistency

NC = no consistency or not relevant to donor human milk banks in Europe

|  | Do your national / local guidelines recommend that milk banks: | Collated Response |
| --- | --- | --- |
| 1 | Recruit donors through a variety of channels, including: written material (in antenatal clinics, maternity shops, etc.), referrals (by donors, physicians, staff), use of mass media, educational programmes | SC |
| 2 | Use clear, non technical language when recruiting | C |
| 3 | Recruit bereaved mothers if they volunteer to donate | SC |
| 4 | Screen donors using oral (informal) interview and/or written questionnaire | C |
| 5 | Require a statement of health by donor’s physician and by donor’s child’s physician | NC |
| 6 | Include the following when screening donors : general health, donor’s child’s information, current or previous medication use, environmental exposure to contaminants, infection exposure, vaccination | SC |
| 7 | Require serological testing of donors for HIV, hepatitis B and hepatitis C with additional screening according to national infection considerations eg HTLV (human T-lymphotropic virus) and Syphilis | C for HIV, hepatits B and hepatitis C. Otherwise SC |
| 8 | Accept antenatal/pregnancy tests as an acceptable alternative to current serology test | NC |
| 9 | Exclude potential donors if drinking more than specified units of alcoholic beverages per specified time period | NC |
| 10 | Exclude prospective donors for: S=smoking, D=recreational drugs, I=infection (with HIV, hepatitis B or C, HTLV (human T-lymphotropic virus), or Syphilis, or increased risk of CJD), M=medication, B=blood transfusion recipient, P=body piercing or tattoos , , V=vegetarian/vegan (doesn’t supplement with B12), X=at risk sexual partner in last 12 m | C (S,D,I,X)  SC(M,B,P)  NC (V) |
| 11 | Exclude prospective donors who spent three months or more in United Kingdom (between 1980 and 1996) or five years or more in Europe from 1980 to present (or risk for Creutzfeldt–Jakob Disease) | NC |
| 12 | Require donors to inform the HMB if there are any changes, and donor is followed up frequently with general health questions | C |
| 13 | Ask donors to stop donating based on the age of the donor’s child | NC |
| 14 | Require informed consent from the donor before accepting her breastmilk | C |
| 15 | Require informed consent from prospective recipient’s mother/guardian before administration of donor milk | C |
| 16 | Train new donors in hand washing and hygiene, expressing milk, storing, cooling, freezing, labeling and transportation of donor milk | C |
| 17 | Provide ongoing support, especially for those who repeatedly donate contaminated milk | C |
| 18 | Advise donors to collect expressed milk rather than drip milk | C |
| 19 | Encourage hand-expressed milk (especially at home) | NC |
| 20 | Accept pump-expressed milk | C |
| 21 | Ensure effective decontamination and disinfection of pump | C |
| 22 | Provide the most effective breast pump for each mother’s specific situation | SC |
| 23 | Emphasize hygiene and hand washing | C |
| 24 | Discourage sharing of breast pumps outside of hospital | C |
| 25 | Recommend that if baby not directly breastfeeding mothers express at least 6, but preferably 8, times every 24 hours | NC |
| 26 | Freeze milk for storage (as soon as possible) at home and within a max of 24 hours | C |
| 27 | Stipulate a maximum storage time length from date of collection before donation to HMB | NC |
| 28 | Require donors to only use containers provided by or approved by the HMB for collection and storage of human milk | C |
| 29 | Accept the collection of multiple expressions in a single refrigerated container | C |
| 30 | Require donors to not add freshly expressed milk to already frozen milk | NC |
| 31 | Provide instructions for defrosting and using breastmilk at home | NC |
| 32 | Place donor human milk immediately in freezer on arrival at the milk bank, or place in a freezer after testing | C |
| 33 | Ensure refrigeration/freezer equipment is used only for HMB milk purposes | C |
| 34 | Monitor freezer temperatures | C |
| 35 | Store raw and pasteurized donor human milk in separate refrigerators / freezers / compartments | C |
| 36 | Emphasise hygienic conditions are in place for processing donor human milk | C |
| 37 | Require staff to wear gloves when handling donor human milk | NC |
| 38 | Thaw frozen raw milk in a refrigerator before pasteurizing | SC |
| 39 | Discard breast milk from donors if it does not meet microbiology or other screening criteria | C |
| 40 | Label donor human milk containers with all relevant information to enable full traceability | C |
| 41 | Should store donor human milk in freezer before pasteurization for a maximum of 3 months | NC |
| 42 | Maintain stated freezer temperature for storage of donor human milk | NC |
| 43 | Maintain stated refrigerator temperature for storage of donor human milk | NC |
| 44 | Store pasteurized donor human milk in freezer for a stated maximum time scale | NC |
| 45 | Store thawed pasteurized donor human milk refrigerated for maximum a stated maximum time scale | NC |
| 46 | Defrost frozen donor human milk using a stated method eg water bath, refrigerator or at room temperature | NC |
| 47 | May issue pasteurized milk that has been freeze dried and vacuum packed if performed in a milk bank in accordance with GMP and all relevant safety procedures | NC |
| 48 | Specify acceptable containers for storing milk (to include food grade materials, glass bottles/containers, plastic bottles/containers, clean non-sterile containers, strong/rupture-free or resistant containers | NC |
| 49 | Specify that milk bags or plastic bags are not recommended for storage | NC |
| 50 | Seal containers with solid lids | NC |
| 51 | Do not overfill containers; fill to about 3/4 full | C |
| 52 | Minimize exposure to sunlight and/or phototherapy lights | C |
| 53 | Ensure that in places with power supply fluctuations, equipment must be connected to generator | SC |
| 54 | Allow pooling of pre-pasteurized breast milk from the same donor; | C |
| 55 | Pool acceptable raw breast milk from different donors | NC |
| 56 | Do not pool milk from different donors | NC |
| 57 | Do not pool milk if it has already been pasteurized | NC |
| 58 | May create multiple batches of donor human milk from one pool | NC |
| 59 | Ensure donor milk remains frozen during transport | C |
| 60 | Set up contract/agreement to maintain conditions needed for transport of donor human milk | SC |
| 61 | May transport donor human milk between milk banks but that additional labeling/tracking may be required | SC |
| 62 | May safely store freshly expressed donor human milk in a cooler for up to 24 hours with frozen gel packs | NC |
| 63 | May use small amounts of dry ice to keep frozen donor human milk at recommended temperatures during transportation | SC |
| 64 | Pack donor human milk tightly in cooler, filling all spaces | NC |
| 65 | Use transport box/bags that should be insulated and easily cleaned | C |
| 66 | Check and record temperatures throughout transport | NC |
| 67 | Require first donation from donor to undergo bacteriological testing | No C |
| 68 | Undertake further bacteriological testing when donor does not seem to guarantee appropriate hygienic conditions | NC |
| 69 | Perform random-sample bacteriological testing before pasteurization | NC |
| 70 | Bacteriologically screen every batch of donor human milk prior to pasteurization | NC |
| 71 | Screen donor human milk for total viable microorganisms | SC |
| 72 | Screen donor human milk for Enterobacteriaceae (gram-negative bacteria) | NC |
| 73 | Screen donor human milk for *Staphylococcus aureus* | SC |
| 74 | Evaluate/screen donor human milk for color, off-flavor, foreign bodies/impurities | SC |
| 75 | Undertake technical verification of Dornic acidity | NC |
| 76 | Undertake technical verification of content/creamatocrit | NC |
| 77 | Undertake quality control checks that include principles of hazard analysis and critical control point (HACCP) | SC |
| 78 | Undertake treatment/pasteurization with a temperature and time of 62.5^o^C for 30 mins i.e. Holder Pasteurisation | C |
| 79 | Rapidly cool heat treated donor human milk | SC |
| 80 | Monitor process and record temperatures during treatment | C |
| 81 | Undertake treatment/pasteurisation with a temperature and time of 62.5^o^C for 30 mins followed by rapid cooling | C |
| 82 | Undertake post-pasteurisation tests | C |
| 83 | Undertake post-pasteurisation tests for every batch of heat treated donor human milk | NC |
| 84 | Test milk for microbial content post-pasteurisation randomly (V=varies) | SC |
| 85 | Test milk for microbial content post-pasteurisation at least once per month, or every 10 cycles, whichever comes first | NC |
| 86 | Discard pasteurised milk with viable microbial content | NC |
| 87 | Discard opened bottles milk that is used for testing | NC |
| 88 | All recipients of donor milk will receive heat - processed milk, unless physician requests raw | NC |
| 89 | Physician is in charge of prescribing or initiating provision of donor milk | NC |
| 90 | Prioritise preterm newborn or low birth - weight newborn for receipt of donor human milk | SC |
| 91 | Dispose of discard donor human milk as with any other clinical waste | SC |
| 92 | Dispose of donor human milk using drain or put in sewerage system | NC |
| 93 | Dispose of mass quantities of frozen bottles of donor human milk as biological waste | NC |
| 94 | Never microwave to defrost or warm milk | C |
| 95 | Prioritize preterm infants to receive milk expressed during first 4 weeks | SC |
| 96 | Match gestational ages of recipient and donor milk expressed | NC |
| 97 | Track donor human milk from donor to recipient hospital/recipient | NC |
| 98 | Record the following when tracking donor human milk: freezer temperatures, refrigerator temps, pasteurisation processes, stock control, dates of expression, defrost time, administration, expiration | SC |
| 99 | All donor human milk and containers should be labeled at each stage | C |
| 100 | Keep records of donors, milk and containers before and after pasteurisation, batch details | SC |
| 101 | Should require receiving hospital to record/document how donor human milk is used | C |
| 102 | Keep tracking and traceability records for a stated minimum time | NC |
| 103 | Undertake a mock recall within specified intervals | NC |
| 104 | Employ staff that include a medical officer, coordinator, infant feeding specialist or nutritionist, nurse, infection control specialist | NC |
| 105 | Update and train staff at a specified frequency  Do your national / local guidelines recommend that milk banks: | NC |
| 106 | Provide staff training that includes hygiene, quality control, safety, and tracking/tracing, technical procedures (collection, storage, pasteurisation), infant nutrition, legal and ethical regulations) | SC |
| 107 | Employ staff be trained in the promotion of breastfeeding | SC |
| 108 | Ensure staff have health checks and are immunised | C |
